# Timing and Weather Offer Alternative Mitigation Strategies for Lowering Bat Mortality at Wind Energy Facilities in Ontario

**DOI:** 10.3390/ani11123503

**Published:** 2021-12-08

**Authors:** Kelly A. Squires, Bethany G. Thurber, J. Ryan Zimmerling, Charles M. Francis

**Affiliations:** 1 Tau Ecology Research Services, Courtenay, BC V9N 0C8, Canada; squireskellya@gmail.com; 2 Canadian Wildlife Service, Environment and Climate Change Canada, 4905 Dufferin St., North York, ON M3H 5T4, Canada; 3 Canadian Wildlife Service, Environment and Climate Change Canada, 351 St. Joseph Boulevard, Gatineau, QC K1A 0H3, Canada; ryan.zimmerling@ec.gc.ca; 4 Canadian Wildlife Service, Environment and Climate Change Canada, 1125 Colonel By Drive, Ottawa, ON K1S 5B6, Canada; charles.francis@ec.gc.ca

**Keywords:** bat activity, eastern red bat, *Eptesicus fuscus*, hoary bat, *Lasionycteris noctivagans*, *Lasiurus borealis*, *L. cinereus*, mortality, silver-haired bat, southwestern Ontario

## Abstract

**Simple Summary:**

Wind farms represent one of the largest sources of anthropogenic mortality for bats. Wind proponents attempt to mitigate these effects via operational curtailment, such that wind energy is not produced on nights with low wind speeds during the late summer and fall. Our study modeled bat activity and mortality on two timescales (nightly and seasonally) and in response to a range of weather variables. We showed that bat mortality risks could be lowered and opportunities for wind energy production can be increased by focusing curtailment efforts to the periods of the night and year when bats are most active and by considering a wider range of weather variables, compared to standard curtailment strategies.

**Abstract:**

Relatively high mortality of migratory bats at wind energy facilities has prompted research to understand the underlying spatial and temporal factors, with the goal of developing more effective mitigation approaches. We examined acoustic recordings of echolocation calls at 12 sites and post-construction carcass survey data collected at 10 wind energy facilities in Ontario, Canada, to quantify the degree to which timing and regional-scale weather predict bat activity and mortality. Rain and low temperatures consistently predicted low mortality and activity of big brown bats (*Eptesicus fuscus*) and three species of migratory tree bats: hoary bat (*Lasiurus cinereus*), eastern red bat (*L. borealis*), and silver-haired bat (*Lasionycteris noctivagans*). Bat activity occurred in waves with distinct peaks through the season; regardless of seasonal timing, most activities occurred in the first half of the night. We conclude that wind energy facilities could adopt a novel and more effective curtailment strategy based on weather and seasonal and nocturnal timing that would minimize mortality risks for bats while increasing the opportunities for power generation, relative to the mitigation strategy of increasing cut-in wind speed to 5.5 m/s.

## 1. Introduction

Significant numbers of bats are killed at wind energy facilities, leading to concerns that this source of anthropogenic mortality may cause population declines in some species [1,2,3,4,5,6,7,8]. In much of Canada and the USA, three migratory species that commonly roost in trees (“migratory tree bats”) account for more than 75% of all fatalities: hoary bat (*Lasiurus cinereus*), eastern red bat (*Lasiurus borealis*), and silver-haired bat (*Lasionycteris noctivagans*), although significant numbers of little brown myotis (*Myotis lucifugus*), federally endangered in Canada, have also been killed at some wind energy facilities [4,9,10]. While mortality due to wind turbines represents only a small percentage of the total mortality caused by collisions for migratory birds [11,12], wind turbines are one of the largest sources of anthropogenic mortality for bats [1,13].

In response to the high rate of mortality at wind energy facilities, post-construction mitigation is required in many jurisdictions. In general, there are negative relationships between bat activity and mortality levels with wind speed [10,14] and bats are likewise less likely to collide with slow-moving or stationary blades [15]. Accordingly, a common mitigation strategy is to curtail turbine blades, so that they spin more slowly below a certain wind speed threshold by increasing the cut-in speed, the minimum wind speed above which turbine blades spin to generate electricity, and by feathering the pitch of the blades to align parallel to winds [16,17]. The majority of bat mortality has been reported in the late summer and fall [10], which is the same period for which Ontario-based wind energy facilities implement operational curtailment when post-construction monitoring data indicate that a given project must do so [18]. Curtailment based on wind speeds reduces, but does not eliminate, bat mortality at wind energy facilities [19,20]. Experimental studies show that reductions in mortality range from about 50–70% (e.g., 50–70% in Alberta [16]; 47% in West Virginia [21]; 62% in Vermont [22]; 63% throughout North American wind farms [23]).

The variability in the efficacy of curtailment based on turbine cut-in wind speed and industry impetus to optimize energy production has resulted in the development of alternative “smart curtailment” strategies to mitigate bat mortality [24]. Such approaches curtail turbine operations using real-time detection of bat calls near turbines combined with weather conditions. One test of this approach showed an 84.5% reduction in mortality for all bat species and a 91.4% reduction for *Myotis* spp., with a 48% reduction in the time turbines were curtailed compared to a wind speed threshold curtailment approach [25]. In the absence of an ability to implement smart curtailment, more optimal mitigation may be achieved based on spatial or temporal patterns in bat activity. For example, curtailment implemented in the first half of the night has been shown to significantly reduce bat mortality, while curtailment implemented in the second half of the night had less effect, implying that bats are more active in the first part of the night [26]. Strong relationships between bat activity and weather could also be used to apply additional curtailment strategies that seek to minimize mortality while maximizing energy production [19,27,28].

Our study tested whether timing, regional-scale weather variables, and moon phase predicted bat activity and mortality throughout southwestern Ontario to understand whether the province’s mitigation strategy can be further improved to reduce bat mortality and, potentially, to increase opportunities for wind power production. The province of Ontario produces the most wind power of any Canadian province or territory [29], and Ontario wind energy facilities have one of the highest rates of bat mortality [4]. Wind energy facilities in this province that are found to exceed a mortality threshold of 10 bats/turbine/year during a mandated period of post-construction mortality monitoring are required to implement a 5.5 m/s cut-in speed from sunset to sunrise between 15 July and 30 September for the lifetime of the project [18]. We analyzed mortality data throughout the mandated period of post-construction monitoring in Ontario (1 May to 30 October) to determine whether the regulated mitigation window includes seasonal peaks in mortality. We analyzed activity data collected from late summer to fall, since we were primarily interested in nocturnal timing and weather factors that may explain higher mortality risk during peak migration.

## 2. Materials & Methods

### 2.1. Bat Mortality

Bat mortality data were collected at 10 wind energy facilities throughout southwestern Ontario from 1 May to 30 October between 2014 and 2018 using methods outlined in [18]. We used 1–3 years of data from each facility, which ranged in size from three to 140 turbines, of which 3–43 turbines were searched. Only data from years when facilities were not mitigating were included in analyses to avoid confounding mitigating effects with factors influencing bat mortality. The landscape at each of the 10 facilities was primarily agricultural, dominated by corn and soybean crops.

While conducting the mortality searches, technicians estimated the time since death (in days) that had passed since the night of collision for each carcass found based on carcass condition, including the color and consistency of blood and the onset of rigor mortis. Only bats estimated to have died the night before were used in the analyses, as it is difficult to accurately estimate time since death at intervals greater than this. Estimates of daily mortality were calculated from the carcass counts using the Ontario Ministry of Natural Resources (OMNR; this ministry is now referred to as the Ontario Ministry of Northern Development, Mines, Natural Resources, and Forestry) equation for estimated mortality. The OMNR mortality estimator includes correction factors for percent area searched, searcher efficiency, scavenger removal, and the proportion of turbines that were searched [18]. These daily mortality estimates were related to seasonal time and weather variables.

### 2.2. Bat Activity

Ultrasonic SM2 BAT+ acoustic recorders (Wildlife Acoustics, Concord, MA, USA) were placed along the eastern shore of Lake Huron and the northern shore of Lake Erie. Recorders were deployed along fence rows between agricultural fields from 9 August and remained in place up to 15 October 2012 to 2014. Microphones were mounted 1 m above the ground and the recorders were programmed to record continuously from 19:00 h to 06:00 h EST. Data were downloaded every third night.

An analyst used an automated process in Kaledioscope Pro (Wildlife Acoustics, Concord, MA, USA) to identify echolocation calls to species or species groups. Species identification was confirmed by manually reviewing spectrograms. Some echolocation calls could not be identified to species, primarily due to recording quality. Recording quality can be affected by distance from the microphone, environmental factors (e.g., humidity, temperature, or nearby surfaces that cause echoes), or bat abundance [30].

Activity was indexed as the number of echolocation “passes” that could be assigned to a species, where a pass was defined as a single call or a group of multiple calls separated from other calls by an interval substantially greater than the interval between calls within a pass. The total count of passes per hour per recorder was used as the measure of activity (9 August to 15 October). Some researchers suggest that an index of detection/non-detection per time interval is more representative of activity levels compared to counts of bat passes since a single bat may be responsible for multiple passes [31]. However, we found that models fit better to count data while providing similar inference as detection/non-detection data.

### 2.3. Predictions and Covariates in Models

We predicted that bat activity and mortality would be positively related to air temperature and humidity [32,33] and negatively related to air pressure [33], rain [32,34], wind speed [10,32,35], and moon illumination [14].

Hourly data for wind speed and direction, air temperature, rain, humidity, and barometric pressure were compiled from Environment and Climate Change Canada (ECCC) weather stations, positioned 11–42 km away from acoustic recorders and 6–21 km from wind turbines [36]. The closest ECCC weather station to a given acoustic recorder and wind energy facility was used to inform the weather conditions at that location. For mortality analyses, wind speed (measured at 10 m above ground level at the ECCC stations) was converted to wind speed at the average turbine hub height, 90 m above ground level, using the method described by [37].

Moon illumination ($E_{v}$ measured in lux) was calculated using the methodology proposed by [38]. We used the R package “suncalc” to derive the required inputs for the moon’s elongation angle, the earth to moon distance, and the angle between the earth and moon. Illumination was calculated on an hourly basis for activity analyses and on a nightly basis for mortality analyses.

Given the concomitant decline of bat activity and temperature with seasonal time, we related bat activity and mortality to deviations of nightly average temperature from the average of nightly temperatures over the previous 20 years. By including a term for temperature in this way, we avoided redundancy in temperature and time as predictors.

Bat activity was related to hourly weather conditions, while bat mortality was related to weather conditions summarized across entire nights (19:00 h to 06:00 h EST). For mortality, nightly averages for wind direction (calculated using R package “Circular”), wind speed, barometric pressure, hourly intervals with rain, and moon illumination were included as terms in the models. Wind speed and direction were combined and included in two additional variables to test whether bats responded to conditions that promoted or opposed fall migration. The proportion of hours with northerly winds were included to test whether bats responded to favorable wind conditions for the fall migration. The proportion of hours with southerly winds that exceeded 6.5 m/s were used to test whether bats responded to wind conditions unfavorable for the fall migration. Wind speed was used to index unfavorable conditions under the assumption that “unfavorable” only occurred with winds above 6.5 m/s. A categorical variable to represent light or dark conditions was added to activity models, where conditions were considered to be dark in the hours after civil twilight.

### 2.4. Statistical Analyses

All statistical analyses were performed using R (R Version 3.0.0, www.r-project.org, accessed on 31 December 2018). We constructed *a priori* models to test our hypotheses regarding temporal and weather variables. We constructed models so that collinear covariates with variance inflation factors > 3 were not included in the same model [39]. All continuous predictor variables were standardized with a mean of 0 and a standard deviation of 1 [40].

Estimated mortality for all species combined and separately for each species per night was related to date and weather variables using hierarchical generalized additive models (HGAM), which included a smoother on the night of the year for each wind energy facility fit with thin-plate regression splines and Poisson error [41,42]. We compared 34 a priori models (Table S1, available online in the Supporting Information) that represented our hypotheses regarding weather conditions that were favorable and unfavorable for bat migration and foraging. We considered moon illumination, wind speed and direction to be important factors for migration flights, whereas that temperature, pressure, and humidity were more likely to influence foraging conditions, while rain likely influenced both.

To relate bat activity to time and weather, we compared the same 34 a priori models (Table S2, available online in the Supporting Information) that represented our hypotheses regarding weather conditions that were favorable or unfavorable for bat migration and foraging. We related counts of passes per hour for all species combined and for individual species at the 12 acoustic sites to time and weather variables using HGAMs, with a smoother per site on the date and a global smoother on the hour of night fit with thin-plate regression splines and negative binomial error.

To assess support among candidate models, we used Akaike’s information criterion (AIC) [43]. HGAM models were compared using AIC since models did not differ in random structure [44]. We considered models within two AICc units of the model with the lowest AICc to be competitive, but we considered nested models to be competitive only if additional parameters improved the log likelihood and contained informative parameters with 85% confidence intervals that did not include zero [45]. We report parameter estimates and standard errors from the best models containing informative parameters.

## 3. Results

Across 10 wind energy facilities from 1 May to 31 October 2014 to 2018, technicians found 1079 carcasses of six species; 635 of these were estimated to have died the night before. Ninety-six percent were of the three species of migratory tree bats and the big brown bat, closely matching mortality results elsewhere [33,46]. The little brown myotis and tri-colored bat (*Perimyotis subflavus*) comprised only 4% of carcasses. Mortality rates were highest for hoary bat (3.05 ± 0.61 (SE) bats/turbine/year), followed by the silver-haired bat (2.68 ± 0.74), eastern red bat (1.36 ± 0.34), and big brown bat (1.42 ± 0.61). On average across wind energy facilities and years, 8.33 ± 1.84 bat fatalities occurred per turbine per year. Only 16% of bat mortality occurred in May and June, and 3% in October.

### 3.1. Relationship of Bat Mortality to Seasonal Time and Weather

**Figure 1:**
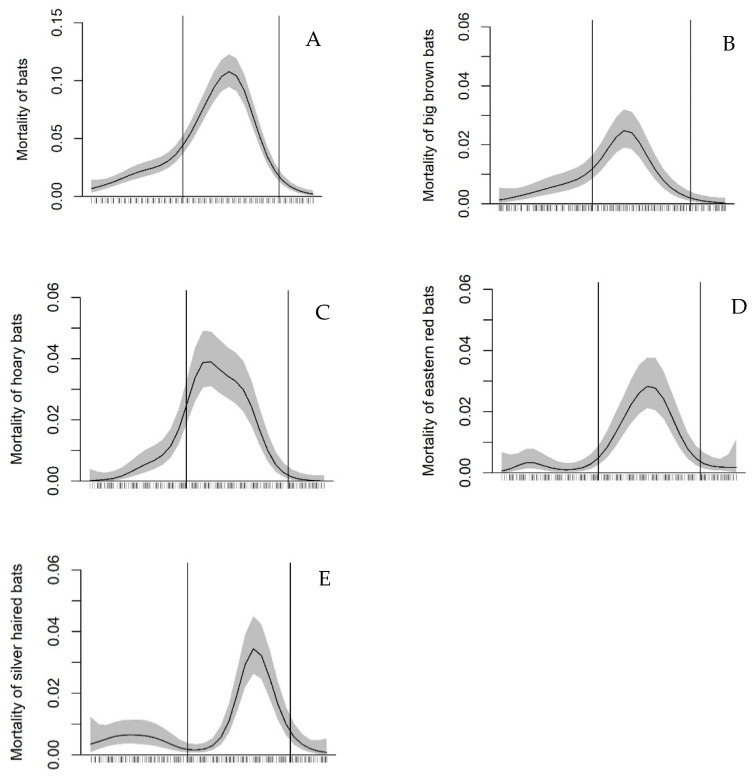
Predicted change in mortality of bats that were estimated to have died the night before (per turbine) with seasonal time (1 May to 31 October 2014 to 2018) at 10 wind energy facilities in southwestern Ontario using generalized additive mixed modeling. Lettered panels show the mortality of all species combined (**A**) and separately by species (**B**–**E**). Vertical bars show the start (15 July) and end (30 September) of the bat mortality mitigation window used at wind energy facilities in Ontario, Canada.

In the analyses relating bat mortality of each species and all species combined to seasonal time and weather variables, model selection resulted in several top models, all of which contained the same informative predictors (Table S1, available online in the Supporting Information). The exception was the silver-haired bat for which the null model fit as well as the model with the lowest AIC. As predicted, bat mortality was positively related to temperature for all species combined and for the hoary and big brown bat. Mortality of all species combined, the big brown and Eastern red bats were negatively related to rain, although for the Eastern red bat, the rain effect was weak since a model with just time was equally supported (Table S1 and Table 1). Only 16% of bat mortality occurred on nights with rain, despite rain occurring on 27% of nights. Mortality of all species combined decreased slightly with the prevalence of strong winds from the south, and hoary bat mortality strongly decreased with increasing wind speed. Predicted mortality showed one peak for all species (Figure 1). Summed across all species, most mortality (75%) occurred during the mitigation window from 15 July to 30 September. For the hoary and big brown bats, a considerable proportion of mortality (17 and 22%, respectively) occurred in late June and early July. Mortality of all species declined across years, a result found in a previous study of these 10 plus an additional 38 facilities in Ontario [7].

### 3.2. Relationship of Bat Activity to Time and Weather

Model comparison using AIC to test hypotheses regarding time and weather as predictors of bat activity resulted in 3 or less top HGAM models per species and for all species combined. Top models contained the same informative predictors, which were most often rain, wind speed, and temperature (Table S2 available online in the Supporting Information). Rain had the strongest negative effect of the weather variables on the activity of all species (Table 2). On 43 nights with rain across all three recording years, only 31% of activity on average was recorded during the hours when it rained (range 7.2–42%). Wind speed also consistently predicted lower activity for all species, the big brown and Eastern red bats were less active with higher moon illumination, while silver-haired bats and all species combined were less active with high barometric pressure. The activity of all species, except the silver-haired bat, was higher when temperatures were above average (Table 2).

**Figure 2:**
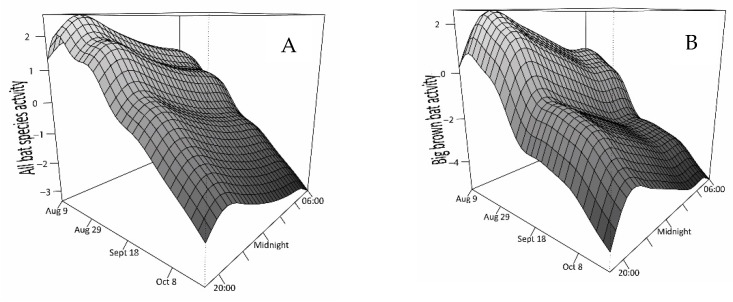

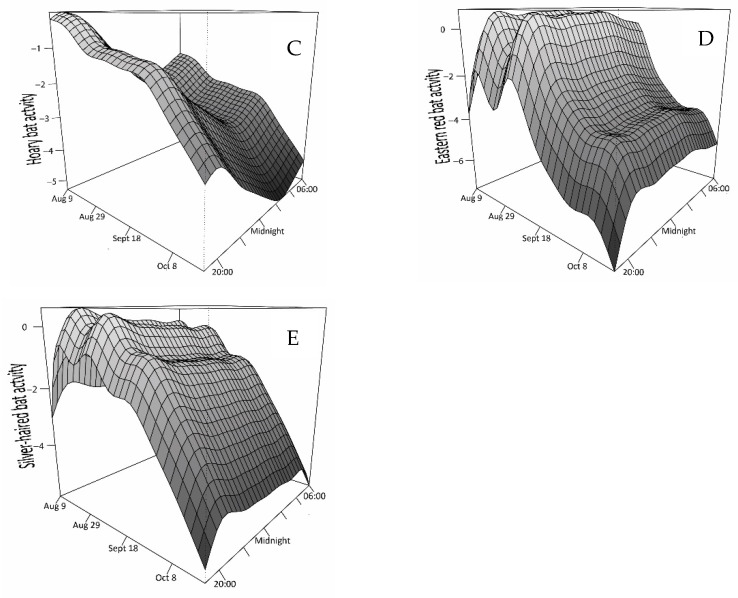
Predicted change (log scale) in bat activity with seasonal (9 August to 15 October) and nocturnal time (19:00–06:00) predicted from generalized additive mixed models fit to counts of passes, measured using echolocation calls identified to species at 12 sites in southwestern Ontario (2012 to 2014). Lettered panels show the activity of all species combined (**A**) and separately by species (**B**–**E**).

Bat activity was predicted to occur in waves in which there were distinct peaks both seasonally and throughout the night (Figure 2). In particular, activity peaked in the first half of the night, though the activity of the hoary bat also showed an increase near dawn. Additionally, all species were recorded during a higher proportion of the nights earlier in the fall. From the earliest dates of acoustic recording in each year to 22 September, bats were recorded for ~40% of the night (range of averages: 30–90%). After 22 September and until the last night with recorded activity on 15 October, bat activity steadily declined and was recorded for on average 20% of the night (range of averages: 10–30%), about 80% of which occurred in the hours prior to midnight (median 77%, range of proportions: 51–89%).

## 4. Discussion

Our results reveal opportunities to improve bat mortality mitigation beyond turbine curtailment based solely on a threshold wind speed. We found that the timing and weather conditions predicted to be favorable and unfavorable for migration flights and for foraging were related to bat activity and mortality. We considered wind conditions, moon illumination, and rain to primarily influence migration flights, while temperature, humidity, air pressure, and rain to influence foraging. We found support for rain, wind speed, and temperature as informative parameters related to the activity and mortality of three species of migratory tree bats in southwestern Ontario. Mortality and activity were lower when it rained, highest with above-average temperatures, and declined with wind speed. These results indicate that migratory bat activity and mortality may be predicted from weather factors that influence both migration flights and foraging conditions. However, it may not be possible to disentangle whether migratory bats are more at risk for turbine collision during weather conditions related to migration flights versus foraging if favorable conditions for migration depend on good foraging opportunities. We found that wind speed and moon illumination also predicted the activity of the non-migratory big brown bat, indicating that these variables may also be related to foraging conditions. Higher winds may limit the availability of aerial insect prey, while moonlight may increase predation risk during foraging and migration flights.

These results corroborate other studies showing timing, temperature, and rain to be consistently strong predictors of the activity of migrating bats [28,47,48]. Relationships with temperature can likely be explained by higher insect availability and foraging opportunities during warm weather and thermoregulatory effects with cooler temperatures. Temperatures below about 10 °C appear to be limiting for bats [22], but there may also be an upper limit to the temperature threshold above which bats reduce their movements. The strong and consistent relationships between bats and temperature offer an opportunity to refine mitigation strategies, such that operational curtailment should be implemented during peak opportunities for migration flights and foraging for bats, but no mitigation may be required when temperatures are lower than those known to limit bat activity [22].

A relatively straightforward strategy to lower bat mortality in Ontario would be to start mitigation earlier in the year. We found that bat mortality and activity occurred in wave-like patterns with distinct peaks, similar to reports for bat activity [34,49]. Our results showed that ~22% of mortality occurred in June and the first half of July before the mitigation window began in Ontario on 15 July. Implementing mitigation earlier in the season would be particularly effective for the big brown and hoary bats, for which the mortality “wave” started earlier in the year compared to the other two species of bats.

Rain was a consistent weather variable related to mortality and activity, and its effect was consistently negative. Rain was the strongest correlate of extended stopovers for migrating silver-haired bats along the Lake Erie coastline [34], and given the high energetic costs of flying with wet fur [50], it follows that bats were less active and therefore subjected to lower mortality risk around wind turbines on nights with rain. Foraging opportunities are also likely fewer during rain, since rain can interfere with echo-location and reduce insect availability (reviewed in [51]). The relationships between the occurrence of rain and bat activity and mortality levels, therefore, may provide an additional means for balancing the conservation responsibilities of mitigating bat mortality through operational curtailment with optimizing power-generating opportunities for wind companies. Wind energy facilities may be able to operate turbines and therefore generate power with a reduced likelihood of mortality for bats when the conditions are sufficiently rainy. Further study is needed to inform mitigation with a quantitative rain threshold that represents limits to bat activity.

Our results are consistent with other studies [14,27] showing a negative effect of wind speed on bat activity, but we found that only the mortality of hoary bats was related to wind speed. A strong relationship between mortality risk and low wind speed should result in consistent outcomes of mitigation based on wind speed. However, the effectiveness of ‘blanket’ mitigation based on wind speed varies widely, from ~40% to 90% mortality reduction [16,17,20]. Some variability may be because some turbines spin, even when curtailed [19]. Another possibility is that the apparent relationship between mortality and low wind speeds is because low wind speeds commonly occur during the peak migration period. For example, the commonly used mitigation strategy based on a cut-in wind speed of 5.5 m/s may result in lower bat mortality simply because this is the median wind speed during bat migration (e.g., [25,35]). Turbines operate for half the time when the curtailment wind speed is the same as the median; although the result is a reduction in mortality risk, it does not indicate a causal relationship between bat activity or mortality and 5.5 m/s wind speed. We note that more recent studies have shown a consistent reduction in bat mortality for every 1 m/s increase in cut-in speed (50% reduction in [23], 33% reduction in [20]). A 40–60% reduction in mortality would be an outcome consistent with mortality being reduced because the turbines are curtailed about half the time.

## 5. Conclusions

We show that timing, temperature, rain, and wind speed predicted bat activity and mortality across four bat species with high mortality rates at wind turbines in southwestern Ontario. Based on these results, we suggest that mitigation to reduce bat mortality from collisions with wind turbines should not be based solely on curtailing when wind speeds are lower than 5.5 m/s. Curtailment during low winds makes economic sense since these conditions result in low electricity production. However, mitigation efficiency can likely be improved with information on weather conditions that represent ecological or physiological limits for bats, particularly thresholds for temperature and the occurrence of rain, to ensure that curtailment is not “wasted” during times when conditions are limiting for bats [19,22,27,28]. We found that timing strongly predicted bat activity and mortality and that bats were more active in the first half of the night, a pattern found in other studies [15,27,28,48]. A potentially more effective mitigation strategy would limit the amount of time that turbine blades rotate during periods of peak activity, while continuing power production during conditions known to limit bat flight or foraging. For example, had turbine blades remained stationary for the first half of every night from mid-August to mid-September, the mortality risk for 65% of recorded activity would have been eliminated. After this period, 80–90% of bat activity occurred before midnight, so power could be generated during the second half of the night with a reduced mortality risk for bats. Nights with rain and low or very high temperatures can provide additional windows for turbines to operate with low risk to bats. Our results suggest that mitigation based on the timing of annual migration and nightly and weather-related patterns in bat activity may provide stronger benefits to both wind energy production and bat populations.

## Figures and Tables

**Table 1:** Parameter estimates of centered and scaled weather variables within HGAM models (Poisson error and log link) relating bat mortality at 10 wind energy facilities in southern Ontario with smoothers fit to the night of the year (1 May to 31 October 2014 to 2018).

Bat Species	Variable	Estimate	SE	*p*
All combined	Year	−0.20	0.06	<0.001
	Temperature	0.16	0.05	<0.01
	Rain	−0.10	0.06	0.08
	Strong southerly wind	−0.12	0.06	0.04
Hoary bat	Year	−0.32	0.10	<0.001
	Temperature	0.31	0.09	<0.01
	Wind speed	−0.43	0.12	<0.001
Big brown bat	Year	−0.35	0.10	<0.01
	Temperature	0.19	0.11	0.09
	Rain	−0.27	0.15	0.09
	Pressure	0.32	0.17	0.07
	Wind speed	−0.13	0.14	0.39
Eastern red bat	Year	−0.07	0.12	0.67
	Rain	−0.25	0.14	0.08

**Table 2:** Parameter estimates of centered and scaled weather variables within HGAM models (negative binomial error and log link) relating bat activity at 12 acoustic recording sites in southwestern Ontario with smoothers fit to the hour of the night and night of the year (9 August to 22 October 2012 to 2014).

Bat Species	Variable	Estimate	SE	*p*
All combined	Temperature	0.32	0.03	<0.001
	Rain	−0.77	0.09	<0.001
	Wind speed	−0.34	0.03	<0.001
	Pressure	−0.05	0.04	0.13
Hoary bat	Temperature	0.29	0.06	<0.001
	Rain	−0.37	0.18	0.05
	Wind speed	−0.18	0.06	<0.01
	Moon	−0.05	0.05	0.36
Big brown bat	Temperature	0.90	0.05	<0.001
	Rain	−0.61	0.13	<0.001
	Wind speed	−0.33	0.04	<0.001
	Moon	−0.19	0.04	<0.001
Eastern red bat	Temperature	0.11	0.05	0.03
	Rain	−0.70	0.17	<0.001
	Wind speed	−0.35	0.06	<0.001
	Moon	−0.18	0.05	<0.001
Silver-haired bat	Temperature	−0.06	0.04	0.12
	Rain	−0.80	0.14	<0.001
	Wind speed	−0.39	0.04	<0.001
	Moon	0.03	0.04	0.43
	Pressure	−0.18	0.05	<0.001

## Data Availability

Acoustic data used in the bat activity analysis were collected by the Government of Canada and may be shared upon request. The bat mortality data are not openly available to the general public as they are property of individual wind farm proponents. They may be made available upon request to the Wind Energy Bird and Bat Monitoring Database committee.

## Supplementary Materials

The following are available online at https://www.mdpi.com/article/10.3390/ani11123503/s1, Table S1: Activity, and Table S2: Mortality.

## References

[1] Cryan P.M. (2011). Wind turbines as landscape impediments to the migratory connectivity of bats. J. Environ. Law.

[2] Arnett E.B., Baerwald E.F., Adams R.A., Peterson S.C. (2013). Impacts of wind energy development on bats: Implications for conservation. Bat Evolution, Ecology, and Conservation.

[3] Hayes M.A. (2013). Bats killed in large numbers at United States wind energy facilities. BioScience.

[4] Zimmerling J.R., Francis C.M. (2016). Bat mortality due to wind turbines in Canada. J. Wildl. Manag..

[5] Frick W.F., Baerwald E.F., Pollock J.F., Barclay R.M.R., Szymanski J.A., Weller T.J., Russell A.L., Loeb S.C., Medellin R.A., McGuire L.P. (2017). Fatalities at wind turbines may threaten population viability of a migratory bat. Biol. Conserv..

[6] Hammerson G.A., Kling M., Harkness M., Ormes M., Young B.E. (2017). Strong geographic and temporal patterns in conservation status of North American bats. Biol. Conserv..

[7] Davy C.M., Squires K., Zimmerling J.R. (2020). Estimation of spatiotemporal trends in bats abundance from mortality data collected at wind turbines. Conserv. Biol..

[8] Friedenberg N.A., Frick W.F. (2021). Assessing fatality minimization for hoary bats amid continued wind energy development. Biol. Conserv..

[9] Johnson G.D. (2005). A review of bat mortality at wind-energy developments in the United States. Bat Res. News.

[10] Arnett E.B., Brown K., Erickson W.P., Fiedler J.K., Hamilton B.L., Henry T.L., Jain A., Johnson G.D., Kerns J., Korford R.R. (2008). Patterns of fatality of bats at wind energy facilities in North America. J. Wildl. Manag..

[11] Calvert A.M., Bishop C.A., Elliot R.D., Krebs E.A., Kydd T.M., Machtans C.S., Robertson G.J. (2013). A synthesis of human-related avian mortality in Canada. Avian Conserv. Ecol..

[12] Zimmerling J.R., Pomeroy A.C., d’Entremont M.V., Francis C.M. (2013). Canadian estimate of bird mortality due to collisions and direct habitat loss associated with wind turbine developments. Avian Conserv. Ecol..

[13] O’Shea T.J., Cryan P.M., Hayman D.T.S., Plowright R.K., Streicker D.G. (2016). Multiple mortality events in bats: A global review. Mamm. Rev..

[14] Cryan P.M., Brown A.C. (2007). Migration of bats past a remote island offers clues toward the problem of bat fatalities at wind turbines. Biol. Conserv..

[15] Horn J.W., Arnett E.B., Kunz T.H. (2008). Behavioral responses of bats to operating wind turbines. J. Wildl. Manag..

[16] Baerwald E.F., Edworthy J., Holder M., Barclay R.M.R. (2009). A large-scale mitigation experiment to reduce bat fatalities at wind energy facilities. J. Wildl. Manag..

[17] Arnett E.B., Huso M.M.P., Schirmacher M.R., Hayes J.P. (2011). Altering turbine speed reduces bat mortality at wind-energy facilities. Front. Ecol. Environ..

[18] Ontario Ministry of Natural Resources [OMNR] (2011). Bats and Bat Habitats: Guidelines for Wind Power Projects.

[19] Arnett E.B., Johnson G.D., Erickson W.P., Hein C.D. (2013). A synthesis of operational mitigation studies to reduce bat fatalities at wind energy facilities in North America. A Report Submitted to the National Renewable Energy Laboratory.

[20] Whitby M.D., Schirmacher M.R., Frick W.F. (2021). The State of the Science on Operational Minimization to Reduce Bat Fatality at Wind Energy Facilities. A Report Submitted to National Renewables Energy Lab.

[21] Hein C.D., Prichard A., Mabee T., Schirmacher M.R. (2013). Effectiveness of an Operational Mitigation Experiment to Reduce Bat Fatalities at the Pinnacle Wind Farm, Mineral County, West Virginia, 2012. An Annual Report Submitted to Edison Mission Energy and the Bats and Wind Energy Cooperative.

[22] Martin C.M., Arnett E.B., Stevens R.D., Wallace M.C. (2017). Reducing bat fatalities at wind facilities while improving the economic efficiency of operational mitigation. J. Mammal..

[23] Adams E.M., Gulka J., Williams K.A. (2021). A review of the effectiveness of operational curtailment for reducing bat fatalities at terrestrial wind farms in North America. PLoS ONE.

[24] North American Clean Energy. http://www.nacleanenergy.com/articles/30944/bat-smart-curtailment-suitability-assessment-and-modeling.

[25] Hayes M.A., Hooten L.A., Gilland K.L., Grandgent C., Smith R.L., Lindsay S.R., Collins J.D., Schumacher S.M., Rabie P.A., Gruver J.C. (2019). A smart curtailment approach for reducing bat fatalities and curtailment time at wind energy facilities. Ecol. Appl..

[26] Young D.P., Nomani S., Tidhar W.L., Bay K. (2011). NedPower Mount Storm Wind Energy Facility post-construction avian and bat monitoring: July-October 2010. Report Prepared for NedPower Mount Story.

[27] Weller T.J., Baldwin J.A. (2012). Using echolocation monitoring to model bat occupancy and inform mitigations at wind energy facilities. J. Wildl. Manag..

[28] Farnsworth A., Horton K., Heist K., Bridge E., Diehl R., Frick W., Kelly J., Stepanian P. (2021). The Role of Regional-Scale Weather Variables in Predicting Bat Mortality and Bat Vocalizations: Potential for Use in the Development of Smart Curtailment Algorithms.

[29] Canada Wind Energy Association Homepage. https://canwea.ca/.

[30] Britzke E.R., Gillam E.H., Murray K.L. (2013). Current state of understanding of ultrasonic detectors for the study of bat ecology. Acta. Theriol..

[31] Miller B.W. (2001). A method for determining relative activity of free flying bats using a new activity index for acoustic monitoring. Acta. Chiropt..

[32] Kerns J., Erickson W.P., Arnett E.B., Arnett E.B. (2005). Bat and bird fatality at wind energy facilities in Pennsylvania and West Virginia. Relationships between Bats and Wind Turbines in Pennsylvania and West Virginia: An Assessment of Bat Fatality Search Protocols, Patterns of Fatality, and Behavioral Interactions with Wind Turbines.

[33] Baerwald E.F., Barclay R.M.R. (2011). Patterns of activity and fatality of migratory bats at a wind energy facility in Alberta, Canada. J. Wildl. Manag..

[34] McGuire L.P., Guglielmo C.G., Mackenzie S.A., Taylor P.D. (2012). Migratory stopover in the long-distance migrant silver-haired bat, Lacionycteris noctivagans. J. Anim. Ecol..

[35] Good R.E., Merrill A., Simon S., Murray K.L., Bay K. (2012). Bat Monitoring Studies at the Fowler Ridge Wind Farm, Benton County, Indiana.

[36] Environment and Climate Change Canada Historical Climate Data. http://climate.weather.gc.ca/.

[37] Bañuelos-Ruedas F., Angeles-Camacho C., Rios-Marcuello S. (2010). Analysis and validation of the methodology used in the extrapolation of wind speed data at different heights. Renew. Sustain. Energy Rev..

[38] Austin R.H., Phillips B.F., Webb D.J. (1976). A method for calculating moonlight illuminance at the Earth’s surface. J. Appl. Ecol..

[39] Zuur A.F., Ieno E.N., Elphick C.S. (2010). A protocol for data exploration to avoid common statistical problems. Methods Ecol. Evol..

[40] Harrison X.A., Donaldson L., Correa-Cano M.E., Evans L., Fisher D.N., Goodwin C.E.D., Robinson B.S., Hodgson D.J., Inger R. (2018). A brief introduction to mixed effects modelling and multi-model inference in ecology. PeerJ.

[41] Wood S.N. (2006). Generalized Additive Models: An Introduction with R.

[42] Pedersen E.J., Miller D.L., Simpson G.L., Ross N. (2019). Hierarchical generalized additive models in ecology: An introduction with mgcv. PeerJ.

[43] Burnham K.P., Anderson D.R. (2002). Model Selection and Multimodel Inference: A Practical Information-Theoretic Approach.

[44] Vaida F., Blanchard S. (2005). Conditional Akaike information for mixed-effects models. Biometrika.

[45] Arnold T.W. (2010). Uninformative parameters and model selection using Akaike’s Information Criterion. J. Wildl. Manag..

[46] Jameson J.W., Willis C.K.R. (2012). Bat mortality at a wind power facility in central Canada. Northwest Nat..

[47] Smith A.D., McWilliams S.R. (2016). Bat activity during autumn relates to atmospheric conditions: Implications for coastal wind energy development. J. Mammal..

[48] Muthersbaugh M.S., Ford W.M., Powers K.E., Silvis A. (2019). Activity patterns of bats during the fall and spring along ridgelines in the central Appalachians. J. Fish Wildl. Manag..

[49] Cryan P.M., Veilleux J.P., Lacki M.J. (2007). Migration and the use of autumn, winter, and spring roosts by tree bats. Bats in Forests: Conservation and Management.

[50] Voigt C.C., Schneeberge K., Voigt-Heucke S.L., Lewanzik D. (2011). Rain increases the energy cost of bat flight. Biol. Lett..

[51] Burles D.W., Brigham R.M., Ring R.A., Reimchen T.E. (2009). Influence of weather on two insectivorous bats in a temperate Pacific Northwest rainforest. Can. J. Zool..

